# Supplementary material for: Differential relieving effects of shikonin and its derivatives on inflammation and mucosal barrier damage caused by ulcerative colitis
Source: PeerJ. 2021 Jan 7;9:e10675. doi: 10.7717/peerj.10675 (PMC7797173; doi:10.7717/peerj.10675)
Supplement: Supplemental Information 1 [file peerj-09-10675-s001.doc]

**Chemicals, Regents and antibodies**

SK (#B21682; CAS NO. 517-89-5; LOT: R10J8F39560), AK (#B50783, CAS NO. 517-88-4; LOT: P05M7F14232), naphthoquinone (#AA18930, CAS NO. 475-38-7), acetyl-SK (#B21508, CAS NO. 24502-78-1; LOT: R21J7F16576) and *β, β*-dimethylacryl-SK (#B24012, CAS NO. 24502-79-2; LOT: P12M7F11131) were ordered from Shanghai YuanYe Bio-Technology Co., Ltd (Shanghai, China). DSS (white powder; CAS NO. 9011-18-1; LOT: Q6182; M.W. = 36000 – 50000 Da) was purchased from MP Biomedicals (Shanghai, China). Mesalazine (#A600043-0050; CAS NO. 89-57-6; LOT: B326BA1390) was purchased from Sigma-Aldrich (Shanghai, China). Murine TNF-α (#ab208348), IL-1β (#ab197742), IL-6 (#ab100712), IL-10 (#ab108870) Elisa assay kits were purchased from Abcam (Cambridge, England). COX-2 (#SBJ-M0847), iNOS (#SBJ-M0041) and MPO (#SBJ-M0329) Elisa assay kits were ordered from Nanjing SenBeiJia Biological Technology Co., Ltd., (Nanjing, China). Primers were purchased from Genscript (Nanjing, China). PMSF (phenylmethanesulfonyl fluoride), RIPA lysis buffers (#P0013B) were purchased from Beyotime Institute of Biotechnology (Haimen, China). BCA protein assay kit (#23227) was purchased from Pierce (Rockford, IL, USA).
