# Supplementary material for: Differential relieving effects of shikonin and its derivatives on inflammation and mucosal barrier damage caused by ulcerative colitis
Source: PeerJ. 2021 Jan 7;9:e10675. doi: 10.7717/peerj.10675 (PMC7797173; doi:10.7717/peerj.10675)
Supplement: Supplemental Information 2 [file peerj-09-10675-s002.docx]

**Supplementary data**

**All of them were characterized by ^13^C NMR, ^1^H NMR, melting properties and MS analysis, which were exactly accord with the structure described.**

**Representative ^1^HNMR spectra**

*5,8-Dihydroxynaphthalene-1,4-dione* ***(Naphthoquinone*,** PubChem CID: 10141***)***

Red powder，Mp: 227°C. ^1^H NMR (600 MHz, CDCl_3_) δ 12.41 (s, 2H, -OH), 7.15 (s, 4H, Ar-H). ^13^C NMR (151 MHz, CDCl_3_) δ 172.85 (s, 1C, 4C, 5C, 8C), 134.65 (s, 2C, 3C, 6C, 7C), 111.87 (s, 9C, 10C). ESI-TOF, calcd for C_10_H_6_O_4_ ([M+H]^+^), 191.03, found 190.96. Anal. Calcd for C_10_H_6_O_4_: C, 63.16; H, 3.18; O, 33.65; Found: C, 63.08; H, 3.20; O, 33.66.

*(R)-5,8-Dihydroxy-2-(1-hydroxy-4-methylpent-3-en-1-yl)naphthalene-1,4-dione* ***(Shikonin*,** PubChem CID: 479503***)***

Red powder，Mp: 147-148°C. ^1^H NMR (600 MHz, CDCl_3_) δ 12.60 (s, 1H, -OH), 12.49 (s, 1H, -OH), 7.20 (t, J = 7.2 Hz, 2H, Ar-H), 7.18 – 7.17 (m, 1H, -C-CH=C), 5.21 (dd, J = 8.0, 6.9 Hz, 1H, -OH), 4.93 – 4.90 (m, 1H, -C-CH=C), 4.12 (dd, J = 14.3, 7.1 Hz, 1H, -O-CH), 2.67 – 2.62 (m, 1H, -CH_2_), 2.39 – 2.33 (m, 1H, -CH_2_), 1.76 (s, 3H, -CH_3_), 1.66 (s, 3H, -CH_3_). ^13^C NMR (151 MHz, CDCl_3_) δ 180.66 (s, 8C), 179.86 (s, 5C), 165.48 (s, 4C), 164.87 (s, 1C), 151.44 (s, 7C), 137.48 (s, 6C), 132.41 (s, 14C), 132.30 (s, 2C), 131.87 (s, 3C), 118.44 (s,13C), 112.03 (s, 9C), 111.55 (s, 10C), 68.35 (s, 11C), 35.68 (s, 12C), 25.97 (s, 15C), 18.10 (s, 16C). ESI-TOF, calcd for C_16_H_16_O_5_ ([M+H]^+^), 289.10, found 288.78. Anal. Calcd for C_16_H_16_O_5_: C, 66.66; H, 5.59; O, 27.75; Found: C, 66.65; H, 5.58; O, 27.72.

*(S)-5,8-Dihydroxy-2-(1-hydroxy-4-methylpent-3-en-1-yl)naphthalene-1,4-dione* ***(Alkanin*,** PubChem CID: 32465***)***

Red powder，Mp: 149°C. ^1^H NMR (600 MHz, CDCl_3_) δ 12.60 (d, J = 4.8 Hz, 1H, -OH), 12.50 (d, J = 5.0 Hz, 1H, -OH), 7.21 (t, J = 7.2 Hz, 2H, Ar-H), 7.19 – 7.17 (m, 1H, -C-CH=C), 5.21 (ddd, J = 8.1, 2.7, 1.3 Hz, 2H, -OH, -O-CH), 4.92 (dd, J = 7.8, 4.3 Hz, 1H, -C-CH=C), 2.68 – 2.63 (m, 1H, -CH_2_), 2.36 (dd, J = 14.8, 7.6 Hz, 1H, -CH_2_), 1.76 (s, 3H, -CH_3_), 1.66 (s, 3H, -CH_3_). ^13^C NMR (151 MHz, CDCl_3_) δ 180.66 (s, 8C), 179.86 (s, 5C), 165.48 (s, 4C), 164.87 (s, 1C), 151.44 (s, 7C), 137.48 (s, 6C), 132.41 (s, 14C), 132.30 (s, 2C), 131.87 (s, 3C), 118.44 (s,13C), 112.03 (s, 9C), 111.55 (s, 10C), 68.35 (s, 11C), 35.68 (s, 12C), 25.97 (s, 15C), 18.10 (s, 16C). ESI-TOF, calcd for C_16_H_16_O_5_ ([M+H]^+^), 289.10, found 288.78. Anal. Calcd for C_16_H_16_O_5_: C, 66.66; H, 5.59; O, 27.75; Found: C, 66.67; H, 5.62; O, 27.71.

*(R)-1-(5,8-Dihydroxy-1,4-dioxo-1,4-dihydronaphthalen-2-yl)-4-methylpent-3-en-1-yl 3-methylbut-2-enoate* ***(β, β*-dimethylacrylshikonin,** PubChem CID: 479499***)***

Red powder，Mp: 103-105°C. ^1^H NMR (600 MHz, CDCl_3_) δ 12.60 (s, 1H, -OH), 12.44 (s, 1H, -OH), 7.18 (s, 1H, -C-CH=C), 7.00 – 6.97 (m, 2H, Ar-H), 6.03 (ddd, J = 18.2, 7.3, 4.0 Hz, 1H, -C-CH=C), 5.79 – 5.77 (m, 1H, -O-CH-), 5.15 (t, J = 7.3 Hz, 1H, -C-CH=C), 2.63 (dt, J = 11.7, 6.6 Hz, 1H, -CH_2_-), 2.48 (dt, J = 14.9, 7.3 Hz, 1H, -CH_2_-), 2.15 (d, J = 10.1 Hz, 3H, -CH_3_), 1.94 (d, J = 0.8 Hz, 3H, -CH_3_), 1.69 (s, 3H, -CH_3_), 1.58 (s, 3H, -CH_3_). ^13^C NMR (151 MHz, CDCl_3_) δ 179.03 (s, 8C), 177.53 (s, 5C), 166.79 (s, 17C), 166.25 (s, 4C), 165.27 (s, 1C), 159.01 (s, 19C), 149.05 (s, 7C), 135.86 (s, 6C), 132.60 (s, 14C), 132.45 (s, 2C), 131.60 (s, 3C), 118.01 (s, 13C), 115.26 (s, 18C), 111.87 (s, 9C), 111.60 (s, 10C), 68.63 (s, 11C), 32.90 (s, 12C), 27.59 (s, 21C), 25.77 (s, 16C), 20.38 (s, 20C), 17.97 (s, 15C). ESI-TOF, calcd for C_21_H_22_O_6_ ([M+H]^+^), 371.14, found 370.10. Anal. Calcd for C_21_H_22_O_6_: C, 68.09; H, 5.99; O, 25.92; Found: C, 68.09; H, 5.98; O, 25.91

*(R)-1-(5,8-Dihydroxy-1,4-dioxo-1,4-dihydronaphthalen-2-yl)-4-methylpent-3-en-1-yl acetate* ***(Acetylshikonin*,** PubChem CID: 479501***)***

Red powder，Mp: 86°C. ^1^H NMR (600 MHz, CDCl_3_) δ 12.59 (s, 1H, -OH), 12.43 (s, 1H, -OH), 7.19 (s, 2H, Ar-H), 6.99 (s, 1H, -C-CH=C), 6.02 (dd, J = 7.2, 4.6 Hz, 1H, -O-CH-), 5.12 (t, J = 7.3 Hz, 1H, -C-CH=C), 2.65 – 2.60 (m, 2H, -CH_2_-), 2.14 (s, 3H, -CH_3_), 1.69 (s, 3H, -CH_3_), 1.58 (s, 3H, -CH_3_). ^13^C NMR (151 MHz, CDCl_3_) δ 178.23 (s, 8C), 176.71 (s, 5C), 169.79 (s, 17C), 167.48 (s, 4C), 166.96 (s, 1C), 148.22 (s, 7C), 136.14 (s, 6C), 132.90 (s, 14C), 132.74 (s, 2C), 131.47 (s, 3C), 117.67 (s, 13C), 111.83 (s, 9C), 111.57 (s, 10C), 69.53 (s, 11C), 32.83 (s, 12C), 25.78 (s, 16C), 21.00 (s, 18C), 17.95 (s, 15C). ESI-TOF, calcd for C_18_H_18_O_6_ ([M+H]^+^), 331.34, found 331.12. Anal. Calcd for C_18_H_18_O_6_: C, 65.45; H, 5.49; O, 29.06; Found: C, 65.40; H, 558; O, 29.03.

**Representative ^1^HNMR spectra**


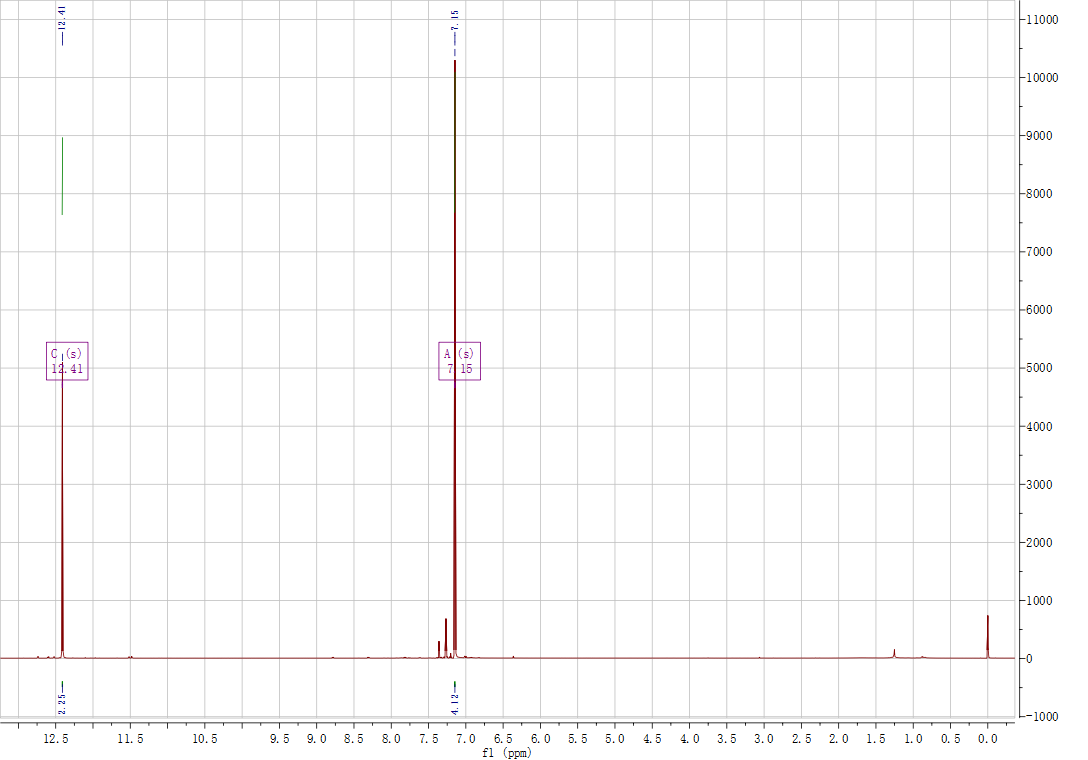


^1^H NMR of Compound **Naphthoquinone**


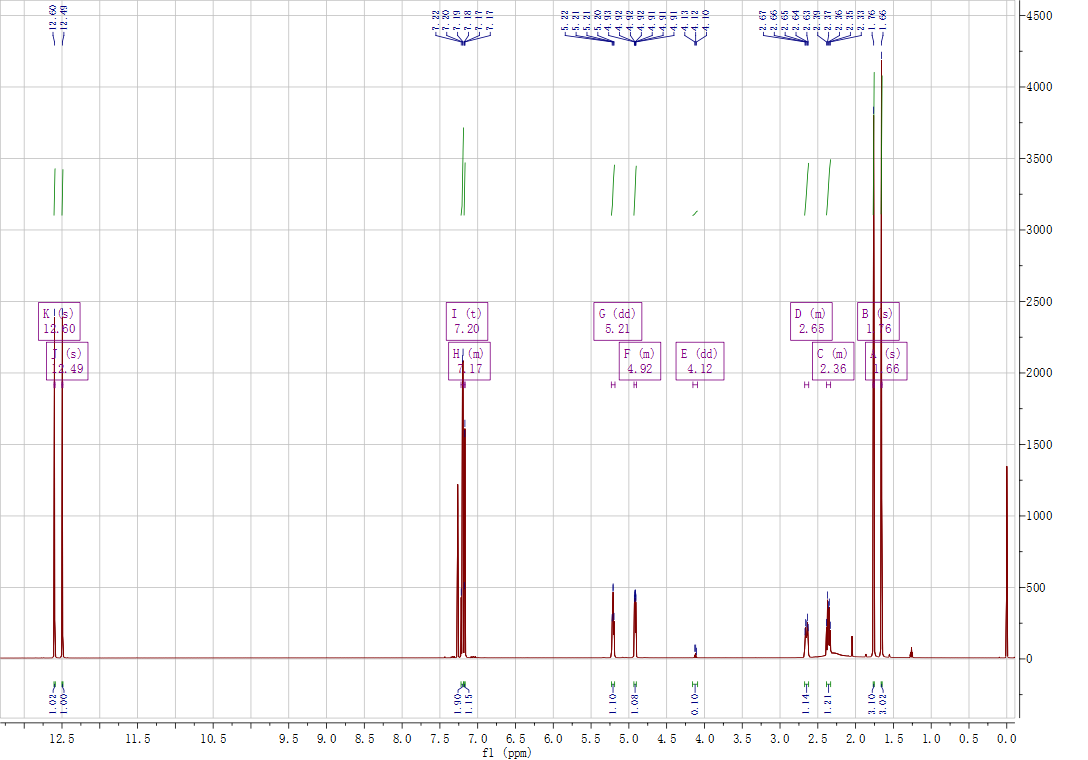


^1^H NMR of Compound **Shikonin**


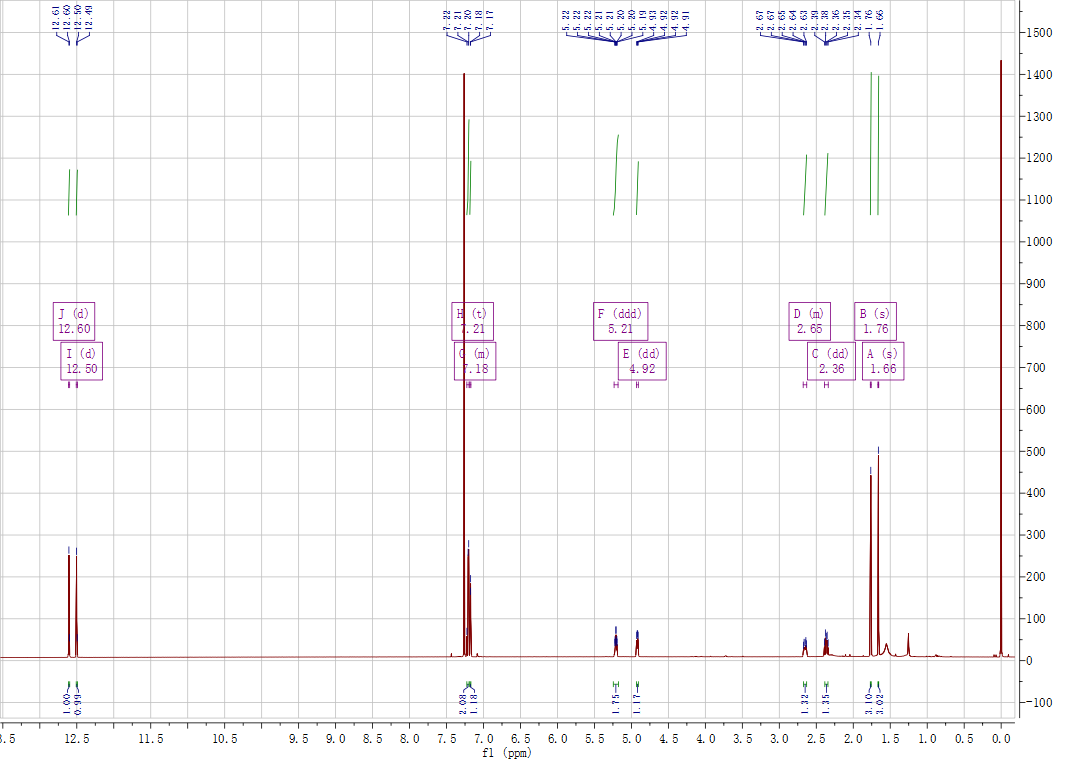


^1^H NMR of Compound **Alkanin**


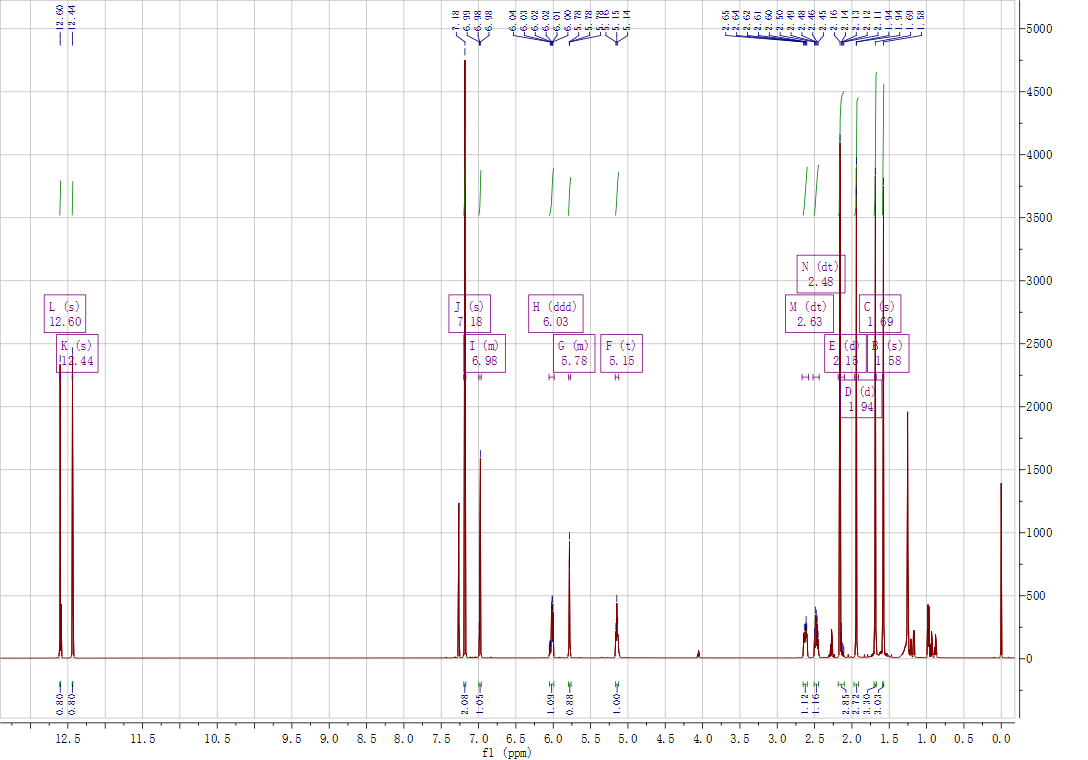


^1^H NMR of Compound ***β, β*-dimethylacrylshikonin**


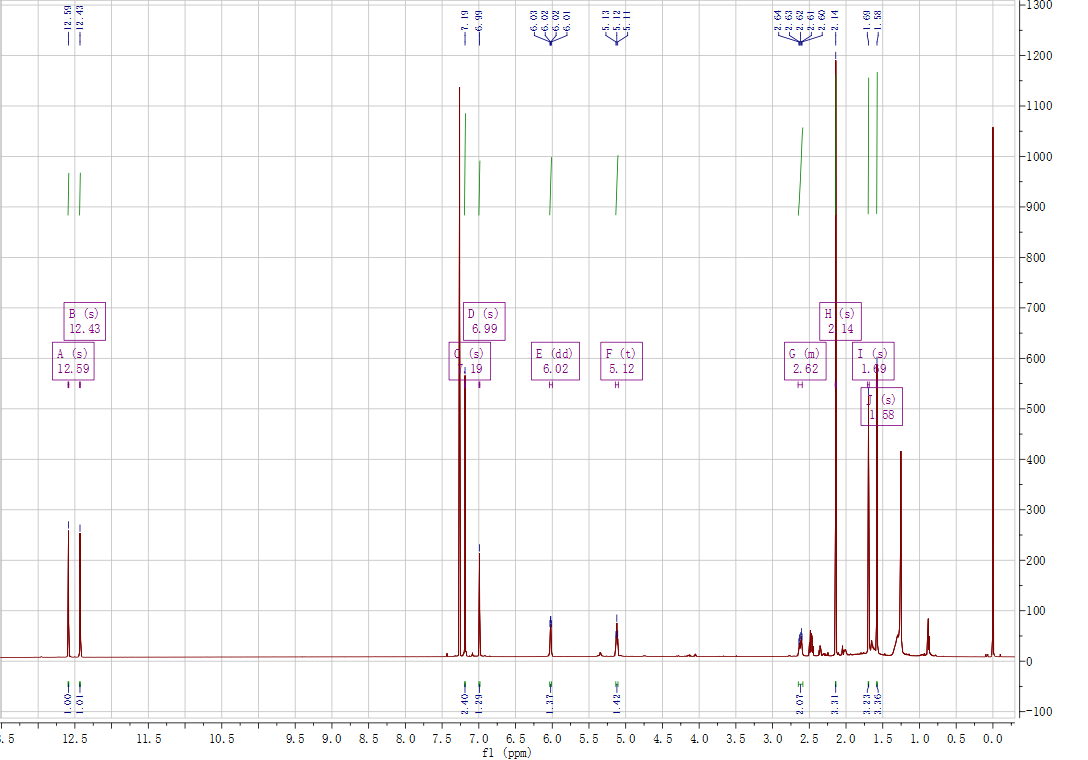


^1^H NMR of Compound **Acetylshikonin**

**Representative ^13^CNMR spectra**

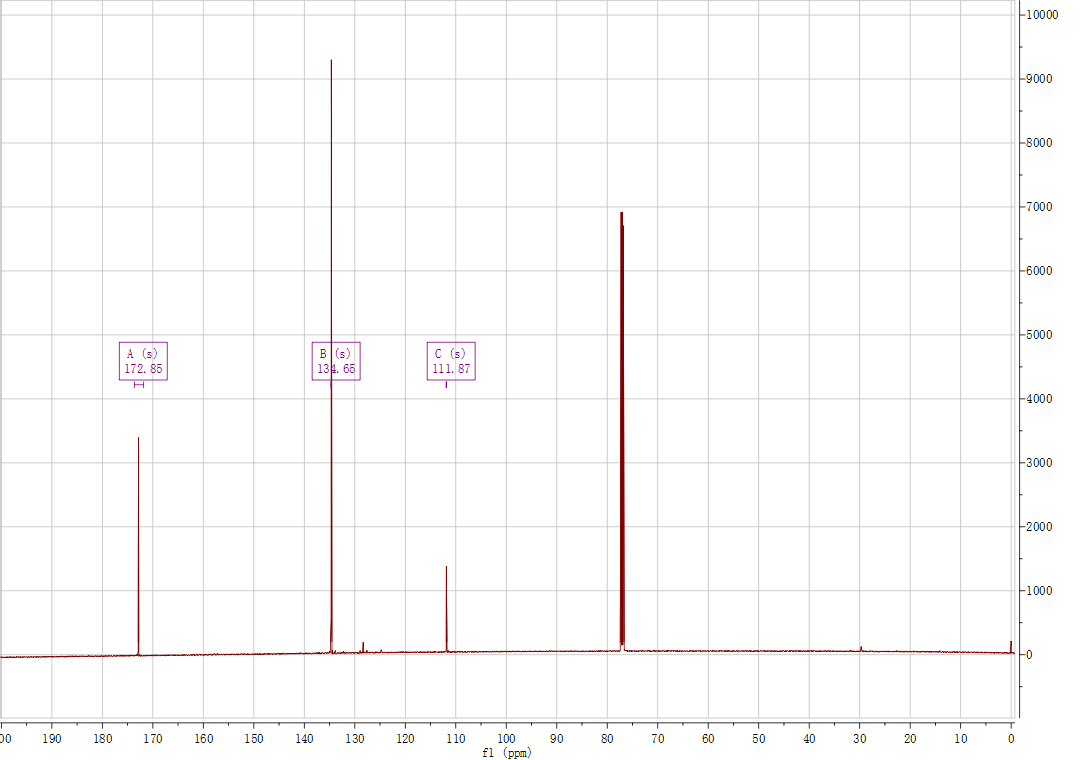


^13^C NMR of Compound **Naphthoquinone**

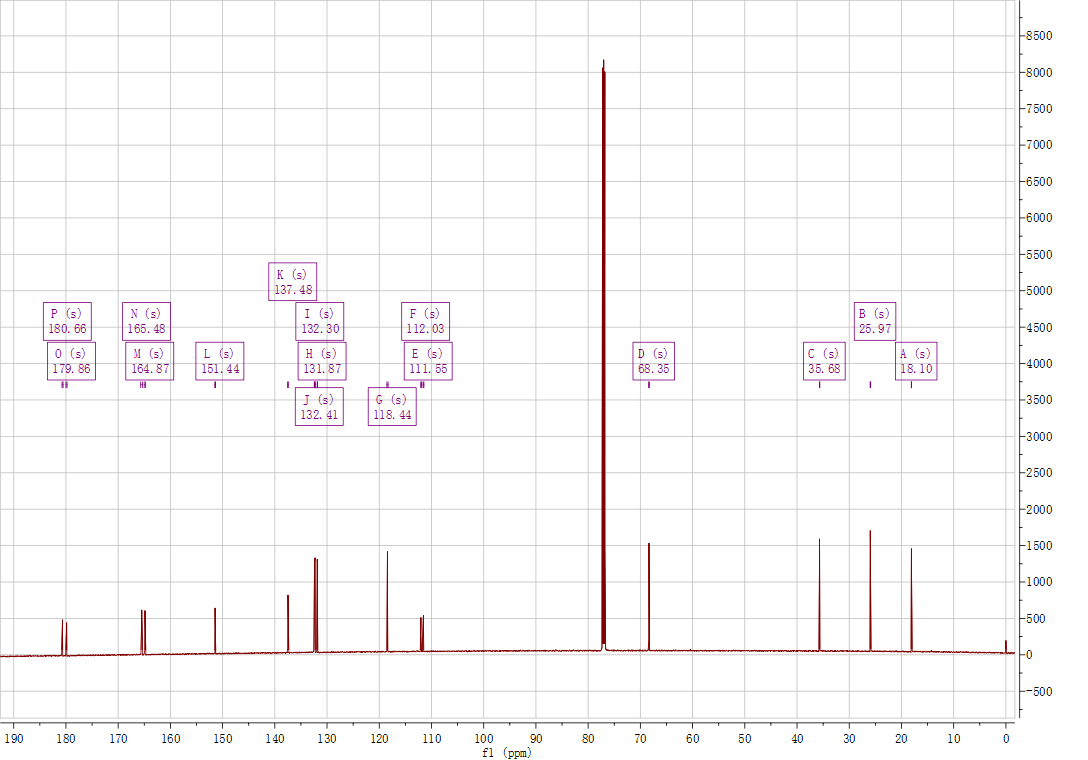


^13^C NMR of Compound **Shikonin**

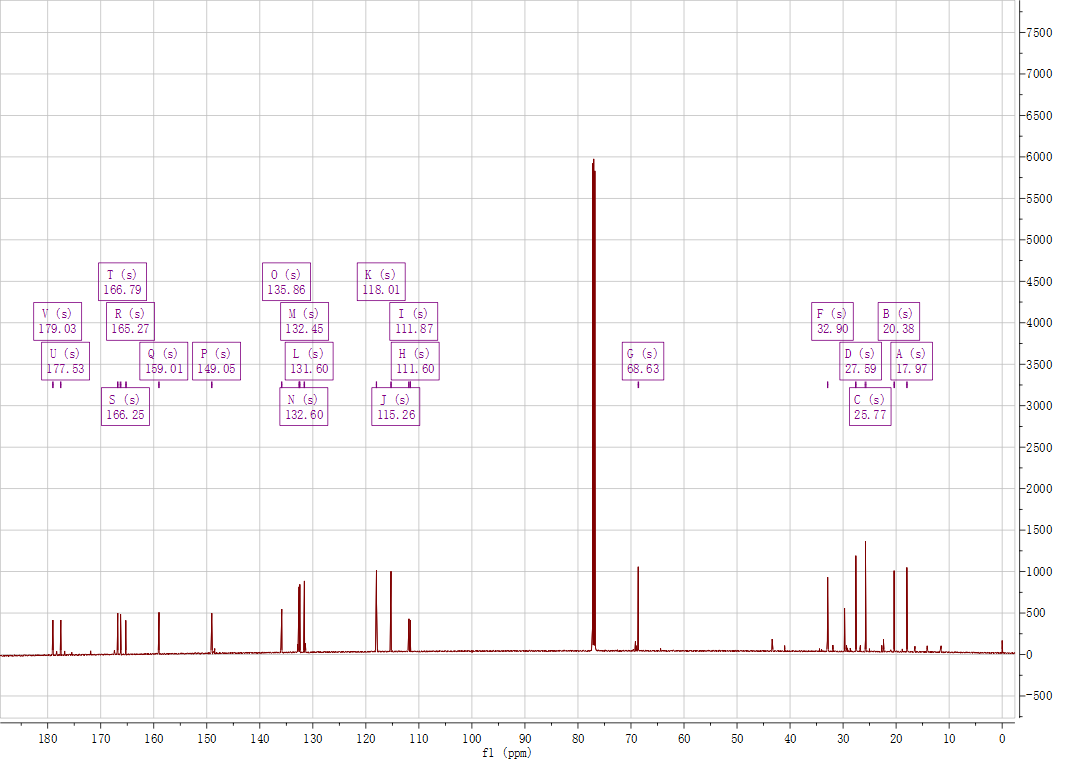


^13^C NMR of Compound ***β, β*-dimethylacrylshikonin**

***
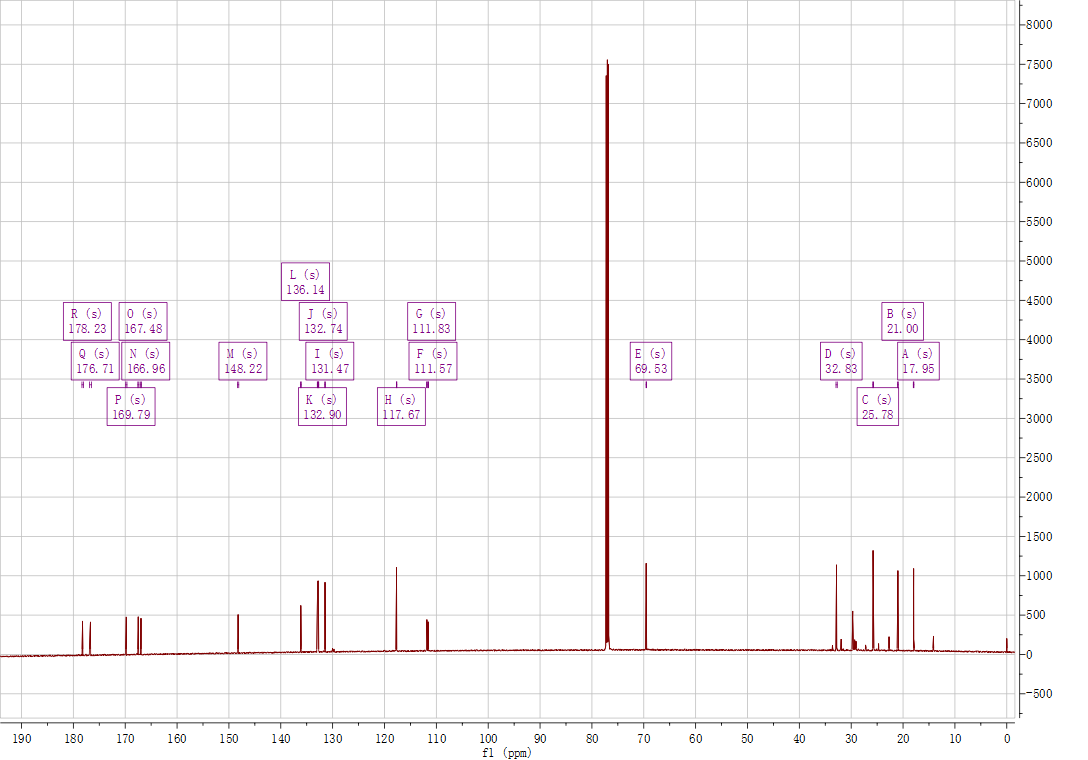
***

^13^C NMR of Compound **Acetylshikonin**
