# Supplementary material for: Differential relieving effects of shikonin and its derivatives on inflammation and mucosal barrier damage caused by ulcerative colitis
Source: PeerJ. 2021 Jan 7;9:e10675. doi: 10.7717/peerj.10675 (PMC7797173; doi:10.7717/peerj.10675)
Supplement: Supplemental Information 11 [file peerj-09-10675-s011.doc]

**Table S1** Information for antibodies.

| **Antibody** | **Vendor** | **Catalogue number** | **Application** | **Dilution** |
| --- | --- | --- | --- | --- |
| iNOS | Enogene | #E1A8233A | WB | 1:500 |
| NF-κB p65 | Enogene | #10745-1-AP | WB | 1:500 |
| GAPDH | Enogene | #10494-1-AP | WB | 1:1000 |
| NLRP3 | Beyotime Biotechnology | #AF2155 | WB | 1:1000 |
| STAT3 | Beyotime Biotechnology | #AF1492 | WB | 1:1000 |
| ZO-1 | Wanlei | #WL03419 | WB | 1:500 |
| Occludin | Wanlei | #WL01996 | WB | 1:1000 |
| Claudin-1 | Wanlei | #WL03073 | WB | 1:1500 |
| ASC | Wanlei | #WL02462 | WB | 1:500 |
| IL-1β | Wanlei | #WL02257 | WB | 1:1000 |
| COX-2 | Wanlei | #WL01750 | WB | 1:1000 |
| p-STAT3 | Wanlei | #WLP2412 | WB | 1:1000 |
| IKB-*α* | Wanlei | #WL01936 | WB | 1:500 |
| phospho -IKB*α* | Wanlei | #WL02495 | WB | 1:500 |
| p-NF-κB p65 (Ser536) | Wanlei | #WL02169 | WB | 1:500 |
| VCAM-1 | Servicebio | #GB11336 | WB | 1:500 |
| Caspase1 | Servicebio | #GB11383 | WB | 1:1000 |
| β-actin | Servicebio | #GB11001 | WB | 1:2000 |
| IKKα | CST | #2682 | WB | 1:1000 |
| IKKβ | CST | #2678 | WB | 1:1000 |
| Rabbit anti-goat IgG HRP | proteintech | #SA00001-2 | WB | 1:5000 |
| Goat anti-mouse IgG (H + L) | Proteintech | #SA00001-1 | WB | 1:5000 |
