# Supplementary material for: Differential relieving effects of shikonin and its derivatives on inflammation and mucosal barrier damage caused by ulcerative colitis
Source: PeerJ. 2021 Jan 7;9:e10675. doi: 10.7717/peerj.10675 (PMC7797173; doi:10.7717/peerj.10675)
Supplement: Supplemental Information 12 [file peerj-09-10675-s012.doc]

**Table** **S2** The standard for the disease activity index (DAI) evaluation.

| **Loss of weight (%)** | **Stool property*** | **Stool occult blood/gross blood stool** | **score** |
| --- | --- | --- | --- |
| 0 | Normal | Normal | 0 |
| 1-5 |  |  | 1 |
| 6-10 | Loose stool | Slight bleeding | 2 |
| 11-15 |  |  | 3 |
| >15 | Liquid stool | Blood in stool | 4 |

*: Normal, formed stool; Loose stool, a paste, semi-formed stool that does not adhere to the anus; Liquid stool, a watery stool that can adhere to the anus.
